# Marked bias towards spontaneous synaptic inhibition distinguishes non-adapting from adapting layer 5 pyramidal neurons in the barrel cortex

**DOI:** 10.1038/s41598-017-14971-z

**Published:** 2017-11-02

**Authors:** Ion R. Popescu, Kathy Q. Le, Rocío Palenzuela, Rebecca Voglewede, Ricardo Mostany

**Affiliations:** 1 Department of Pharmacology, Tulane University School of Medicine, New Orleans, 70112 USA; 2 Neuroscience Program, Brain Institute, Tulane University, New Orleans, 70118 USA; 3 School of Experimental Sciences, Universidad Francisco de Vitoria, Pozuelo de Alarcón, 28223 Madrid, Spain; 4 Brain Institute, Tulane University, New Orleans, 70118 USA

## Abstract

Pyramidal neuron subtypes differ in intrinsic electrophysiology properties and dendritic morphology. However, do different pyramidal neuron subtypes also receive synaptic inputs that are dissimilar in frequency and in excitation/inhibition balance? Unsupervised clustering of three intrinsic parameters that vary by cell subtype – the slow afterhyperpolarization, the sag, and the spike frequency adaptation – split layer 5 barrel cortex pyramidal neurons into two clusters: one of adapting cells and one of non-adapting cells, corresponding to previously described thin- and thick-tufted pyramidal neurons, respectively. Non-adapting neurons presented frequencies of spontaneous inhibitory postsynaptic currents (sIPSCs) and spontaneous excitatory postsynaptic currents (sEPSCs) three- and two-fold higher, respectively, than those of adapting neurons. The IPSC difference between pyramidal subtypes was activity independent. A subset of neurons were thy1-GFP positive, presented characteristics of non-adapting pyramidal neurons, and also had higher IPSC and EPSC frequencies than adapting neurons. The sEPSC/sIPSC frequency ratio was higher in adapting than in non-adapting cells, suggesting a higher excitatory drive in adapting neurons. Therefore, our study on spontaneous synaptic inputs suggests a different extent of synaptic information processing in adapting and non-adapting barrel cortex neurons, and that eventual deficits in inhibition may have differential effects on the excitation/inhibition balance in adapting and non-adapting neurons.

## Introduction

The characterization of synaptic inputs is necessary to establish how pyramidal neurons of different subtypes participate in information processing as well as a starting point to better understand how these neurons are affected by disease. A substantial body of research on intrinsic cell electrophysiology properties and dendritic morphology has shown that neocortical layer 5 (L5) pyramidal neurons belong to two main subtypes referred to as adapting and non-adapting, or thin-tufted and thick-tufted, respectively^1–5^. Because the barrel cortex is a well-established model system used for investigating basic cortical processing and neurological disorders, there is also considerable interest in the EPSCs and IPSCs of pyramidal neurons in this brain area. However, it has not been shown if in the primary somatosensory cortex barrel field (S1BF) these two kinds of neurons differ in their spontaneous postsynaptic currents. The characterization of synaptic inputs will aid in understanding how action potential generation is controlled in adapting and non-adapting neurons, which differ considerably in intrinsic excitability^6^ and have been posited to play different roles in perception and brain state generation^7^. Postsynaptic current (PSC) measurements will indicate which of these pyramidal neuron functions relies more heavily on synaptic inputs. Finally, establishing that there is a baseline difference in spontaneous PSCs between pyramidal neuron subtypes demonstrates the need to categorize the neurons in any study involving comparisons of synaptic inputs between pyramidal neurons from groups containing different subtypes.

Synaptic inputs in the neocortex consist overwhelmingly of glutamatergic EPSCs and GABAergic IPSCs, and are processed by both dendritic morphology and intrinsic electrophysiology properties to determine the timing and strength of action potential output. Three of the intrinsic properties most consistently shown to be differentially expressed in pyramidal neurons are the slow afterhyperpolarization (sAHP), the sag, and the spike frequency adaptation^2–6,8–10^. The sAHP refers to the hyperpolarization occurring 500 ms after several spikes are fired, is apamin insensitive, has Ca^+2^-dependent and Na^+^-dependent components^11–13^, and has been shown to increase coincidence detection in pyramidal neurons during strong background excitation^14^. Sag, referring to the depolarization occurring after the onset of hyperpolarization during sustained injection of hyperpolarizing current, is caused by hyperpolarization-activated cyclic nucleotide-gated (HCN) channels. The opening of these channels reduces cellular excitability, excitatory postsynaptic potential amplitudes, and temporal summation^15–17^. Spike frequency adaptation, the gradual reduction in firing frequency during a constant stimulus, such as constant depolarizing current injection, is mediated by a variety of mechanisms. They include inactivation of Na^+^ channels^18,19^, Ca^+2^-dependent K^+^ currents^20–22^, Na^+^-activated K^+^ current^13^, and M-type K^+^ current^23,24^. Adaptation has been proposed as a mechanism for preventing runaway excitation caused by recurrent excitatory connectivity in the cortex^25^. Several studies have also focused on the role of adaptation in maximizing information transfer^26–30^.

In neocortical pyramidal neurons the co-occurrence of minimal spike frequency adaptation, small sAHP, high sag, and a thick apical dendrite tuft has been demonstrated^3–6,8,9^. Conversely, the co-occurrence of pronounced spike frequency adaptation, large sAHP, low sag, and a thin dendritic tuft has also been shown^3–6,8,10^. Additionally, thick-tufted neurons have been associated with bursting firing patterns and thin-tufted neurons with non-bursting, “regular spiking” patterns, mostly in sharp electrode recordings^10,31–33^.

The fact that pyramidal neurons from the same layer have different electrophysiological and anatomical profiles hints that their synaptic input profiles may also differ. This is also suggested more directly by the effects of synaptic inputs on sag^16^ and on sAHP^34,35^, and by the relationship between synaptic inputs and the effect of spike frequency adaptation^28^.

To establish if spontaneous synaptic inputs differ between L5 pyramidal neuron subtypes in the barrel cortex we performed whole-cell patch clamp recordings in acute slices. We measured intrinsic properties (sAHP, sag, and spike frequency adaptation) as well as synaptic inputs, and we filled a subset of L5 pyramidal cells for morphological analysis. Subsequent unsupervised cluster analysis classified the pyramidal neurons into two clusters of cells, adapting and non-adapting neurons, with significantly different intrinsic properties and dendritic morphologies. We found significant differences in the amounts of synaptic inputs, both excitatory and inhibitory, that these two main subtypes of L5 pyramidal neurons of S1BF receive.

## Methods

### Animals

We used male and female Tg(Thy-1-EGFP)MJrs/J (GFP-M) transgenic mice, 1–6 months of age (94.5 ± 6.0 d, *n* = 36), which present with sparse GFP labeling of L5 pyramidal neurons under the thy-1 promoter^36^. Mice were group housed by gender under a 12 h light, 12 h dark cycle, and had access to nesting material as well as food and water *ad libitum*. Experiments were performed during the light hours of the cycle. All the procedures described in this study were approved by the Institutional Animal Care and Use Committee of Tulane University, and were performed in accordance with the NIH Office of Laboratory Animal Welfare’s *Public Health Service Policy on Humane Care and Use of Laboratory Animals* and *Guide for the Care and Use of Laboratory Animals*.

### Brain slice preparation

Mice received general anesthesia by isoflurane inhalation, after which they were decapitated and the brain was quickly removed and submerged in an iced sucrose solution containing (in mM): 234 sucrose, 2.5 KCl, 25 NaHCO_3_, 1.25 NaH_2_PO_4_, 7 MgCl_2_, 0.5 CaCl_2_, 7 glucose, pH 7.3–7.4, bubbled with 95% O_2_, 5% CO_2_. After 2 min, the brain was blocked in the coronal plane anterior and posterior to the somatosensory cortex. The anterior plane was attached with cyanoacrylate-based glue to a detachable stage, after which the brain was sliced in 350 µm increments on a vibratome while submerged in iced sucrose solution. These coronal brain slices were then incubated 45–60 min at 30 °C in aCSF (in mM): 125 NaCl, 2.5 KCl, 25 NaHCO_3_, 1.25 NaH_2_PO_4_, 2 CaCl_2_, 25 glucose, pH 7.3–7.4, bubbled with 95% O_2_, 5% CO_2_. Afterwards, slices were maintained for at least 1 hour at RT in aCSF prior to being moved to the recording chamber. Slices were allowed to equilibrate for at least 15 min in the recording chamber prior to recording.

### Cell morphology

Neurons were filled with biocytin (0.5% in the patch electrode solution) through the patch pipette during recordings lasting 15–20 min in voltage clamp or current clamp mode. Subsequently, the patch pipette was slowly withdrawn. After overnight fixation in 4% paraformaldehyde, slices were rinsed in PBS and incubated in blocking solution (2% BSA, 5% sucrose, 1% Triton X-100 in PBS) for 1 hour at RT. Slices were then incubated with Streptavidin-Alexa 594 (1/400) in blocking solution overnight at 4 °C. The next day, the slices were thoroughly washed in PBS and mounted using Fluoromount Aqueous Mounting Medium. Images were acquired with an A1Rsi confocal microscope (Nikon Instruments Ltd., Japan) using a 10X, 0.45 NA objective. ImageJ (http://rsb.info.nih.gov/ij/) was used for quantification. The tuft width was defined as the horizontal width of the full dendritic tuft, including the tufts of all primary apical dendrites. Apical dendrite length was obtained by measuring the distance from the intersection of the base of the apical dendrite with an ellipse inscribed in the cell body to the point where the tuft of the primary apical dendrite began. Shaft width was calculated as the mean full width at half maximum (FWHM) of the apical dendrite at 225, 250, and 275 µm from the interception of the major and minor axes defining the aforementioned inscribed ellipse. An apical dendrite was counted as a primary apical dendrite if it bifurcated from the main apical dendrite, and extended toward and formed a dendritic tuft near the pia.

### Electrophysiology

The recording chamber was perfused continuously at a rate of 2 ml/min with aCSF bubbled with 95% O_2_, 5% CO_2_, and warmed to 28–30 °C. Patch pipettes were pulled in three stages on a horizontal puller (Sutter Instruments, Novato, CA, USA) from glass capillaries with ID of 1.2 mm and OD of 1.65 mm (KG-33, King Precision Glass, Claremont, CA, USA). When filled with patch solution (in mM: 70 K-gluconate, 70 KCl, 2 NaCl, 2 MgCl_2_, 10 HEPES, 1 EGTA, 2 MgATP, 0.3 Na_2_GTP, 290 mOsm, pH 7.3 adjusted with KOH), the pipettes had a resistance of 2.5–4.5 MΩ. In some recordings biocytin (0.5%) was included in the patch solution for subsequent morphological analysis. Pyramidal cells were identified by their triangular shape and their apical dendrite or by the expression of GFP. Cells were patch-clamped while visualized with a 40X immersion objective and Dodt gradient contrast in a SliceScope microscope (Scientifica, UK). GFP was visualized with a 470 nm LED passing through a filter set that consisted of an HQ470/40X excitation filter, a dichroic mirror Q495LP, and an HQ525/50 nm emission filter. Recordings were made using a Multiclamp 700B amplifier and a Digidata 1550 digitizer controlled with the Multiclamp Commander program and the pClamp 10 program (Molecular Devices, Sunnyvale, CA, USA). The acquisition frequency was 10 kHz. Voltage clamp traces were Bessel filtered at 2 kHz during acquisition. The bridge was balanced automatically in Multiclamp Commander prior to attempting seal formation. Fast capacitance transients were compensated automatically in Commander upon GΩ seal formation. Recordings were terminated if the access resistance monitored in the Clampex Membrane Test was ≥30 MΩ. The input resistance was calculated in voltage clamp from 5 mV hyperpolarizing steps. The membrane potential was not adjusted for the liquid junction potential. Cells with resting membrane potential positive or equal to −60 mV were excluded from the analysis.

To compute the spike frequency adaptation index, we elicited spikes with a series of 2 s current pulses of amplitude increasing in 5 to 20 pA increments. The index was taken from steps containing 12–16 action potentials (6–8 Hz). The sAHP was measured by eliciting 35 action potentials during 500 ms by injecting current via the patch pipette in 5 ms pulses. The pulse amplitude was adjusted to elicit single action potentials. To study the sag we delivered a series of 2 s hyperpolarizing pulses from resting potential in current clamp. The pulse amplitude was increased in 20–40 pA increments and % sag was calculated from steps in which the maximal hyperpolarization was −80 mV to −90 mV.

IPSCs were recorded as inward currents at −70 mV in the presence of 20 µM 6,7-Dinitroquinoxaline-2,3-dione (DNQX). To record miniature IPSCs (mIPSCs), the voltage-gated sodium channel antagonist tetrodotoxin (TTX) (1 µM) was bath applied until action potentials could no longer be elicited with intracellular injection in current clamp. mIPSCs were recorded subsequently in voltage clamp mode. Simultaneous application of picrotoxin (60 µM) in addition to 20 µM DNQX eliminated all detectable spontaneous PSCs when cells were voltage-clamped at −70 mV (*n* = 3 cells). sEPSCs were recorded at −80 mV in the presence of 40 µM bicuculline methiodide. Application of 40 µM bicuculline and 20 µM DNQX after a control period abolished all detectable spontaneous PSCs when cells were voltage-clamped at −80 mV (*n* = 10). Picrotoxin, bicuculline methiodide, DNQX, and TTX were stored in frozen aliquots at 1,000X the working concentration.

### Electrophysiology data analysis

To determine the spike frequency adaptation index, we measured the interspike interval (ISI) of successive spikes, excluding the first two ISIs. Each ISI was then normalized to the third ISI and plotted as a function of the sequential ISI number. The slope of the linear regression was multiplied by 100 to obtain the adaptation index^6,10^. Cells with irregular, stuttering spiking were excluded. Repetitive bursting was only encountered in 3% of cells recorded, and those cells were excluded. To determine the sAHP, five consecutive sweeps were averaged and the sAHP was measured at 500 ms after the last action potential, relative to the resting membrane potential just before stimulation^6^. % sag = 100*(peak change - steady-state change)/(peak change)^6^. Peak change and steady-state change were measured from the membrane potential just prior to current injection.

Principal component analysis (PCA) and hierarchical clustering were computed using MATLAB (MathWorks, Natick, MA, USA). Values for each variable (spike frequency adaptation index, sAHP, and % sag) from each recording (sample) were scaled (zero mean and unit variance) and centered (subtracting off the mean) before both PCA and pairwise distance between pairs of values were calculated. Singular value decomposition (SVD) algorithm was used to perform PCA. Unsupervised hierarchical clustering was computed using *correlation* as the distance metric and *average* method for linkage of samples. The results from the cluster analysis were further validated using the *clValid* package^37^ on R Statistical Software (The R Foundation for Statistical Computing, Vienna, Austria; Version 3.4.1).

sIPSCs and mIPSCs were detected and measured with MiniAnalysis (Synaptosoft, Fort Lee, NJ, USA), using a detection threshold of 5 pA. The reported IPSC decay time is the time from peak amplitude to 37% of peak amplitude. Rise time was measured from rise onset time to peak time.

### Statistics

Data are provided in text, figures, and tables as mean ± SE. Statistical differences between groups were determined using the Student’s *t*-test. Paired *t*-test was used for within-cell comparisons. Unpaired two-sample *t*-tests assuming equal variances or assuming unequal variances were used accordingly after applying the *F*-test two-sample for variances in each case. One-way ANOVA followed by Bonferroni *post hoc* test was used to compare adapting, thy1-GFP-positive (GFP+), and non-adapting thy1-GFP-negative (GFP−) neurons. Statistical significance was set at *p* < 0.05. The datasets generated during and/or analyzed during the current study are available from the corresponding author upon reasonable request.

## Results

### Intrinsic electrophysiology and clustering of L5 pyramidal neurons

**Figure 1:**
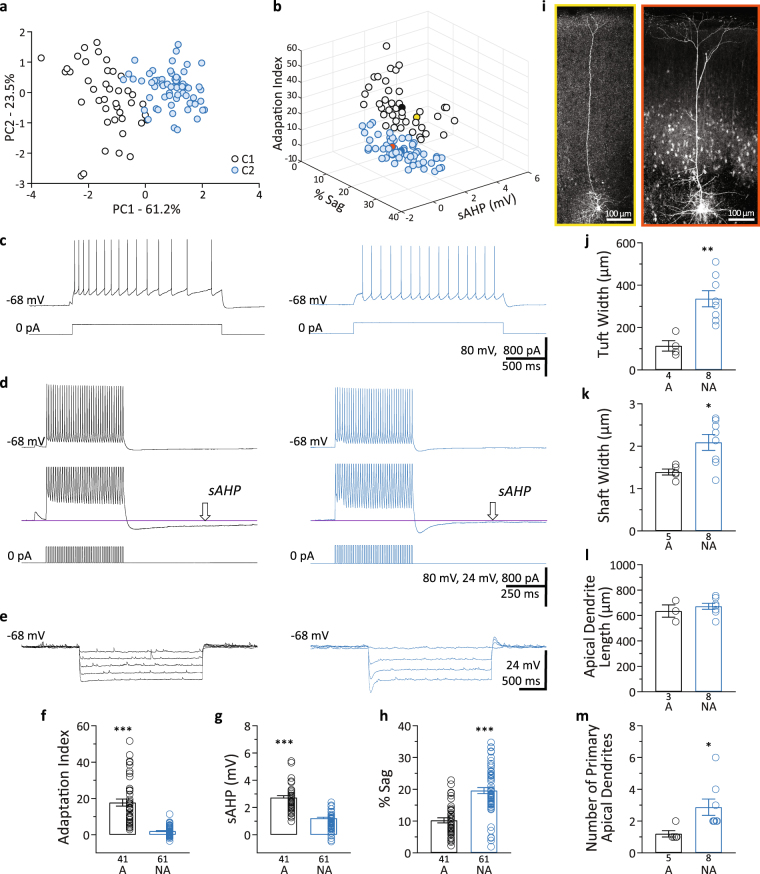
Identification of adapting and non-adapting L5 pyramidal neurons. (**a**) PCA score-plot for the first two principal components for each L5 pyramidal neuron showing the cell clusters detected. Cluster 1 (C1) corresponds to the adapting neurons and C2 corresponds to the non-adapting neurons. (**b**) 3D scatter plot of the intrinsic properties, adaptation index, % sag, and sAHP, used for the clustering analysis. White circles, adapting neurons; blue circles, non-adapting neurons; solid black and blue circles correspond to the centroids of the two clusters; yellow circle, adapting cell illustrated in panel i-left; orange circle, non-adapting cell illustrated in panel i-right. (**c**) Examples of action potential trains (upper traces) fired in response to intracellular current injection (lower traces). Left upper trace shows considerable spike frequency adaptation (Adaptation Index = 22.1). Right upper trace shows minimal adaptation (Adaptation Index = 0.5). (**d**) Upper traces, examples of sAHP from an adapting neuron (left) and a non-adapting neuron (right). Middle traces, Y-axis expansion of upper traces. The amplitude of the sAHP is measured at the arrow. Lower traces, intracellular current injection. (**e**) Examples of membrane potential sag in response to hyperpolarizing current steps from an adapting (left) and a non-adapting (right) neuron. Larger current steps were used in (**e**) in the neuron on the right. All representative traces belong to the same adapting (left traces) or non-adapting (right traces) neuron (**c**–**e**). (**f–h**) Summary of intrinsic properties for the adapting (A) and non-adapting (NA) groups of L5 pyramidal neurons. (**f**) Spike frequency adaptation index; (**g**) sAHP; and (**h**) %sag. (**i–m**) Morphological analysis of adapting and non-adapting L5 pyramidal neurons in S1BF. (**i**) Representative microphotographs of biocytin-labeled neurons. Left/yellow border, adapting neuron; Right/orange border, non-adapting neuron. (**j**) Tuft width; (**k**) Shaft width; (**l**) Length of primary apical dendrites; (**m**) Number of primary apical dendrites. Data presented as mean ± standard error. *p < 0.05; **p < 0.01; ***p < 0.001.

To distinguish between L5 pyramidal neuron subtypes in S1BF we quantified three intrinsic electrophysiology parameters whose differential expression in neocortical pyramidal neurons has been extensively documented: sAHP, sag, and spike frequency adaptation index^2–6,8–10^. These active membrane properties play a role in determining action potential output in response to synaptic inputs. We applied PCA to the values obtained for the measured parameters from each individual recording (*n* = 102 cells). The first two principal components (PC1 and PC2) explained 61.2% and 23.5% of the total variance, respectively (Fig. 1a). The component loadings indicated that adaptation index (−0.61) and % sag (0.61) were negatively correlated as previously reported^3,4,6,8^, and were the variables that, making similar contributions, influenced PC1 the most. Unsupervised cluster analysis and further validation revealed two clusters (cluster 1, C1 and cluster 2, C2) of neurons (Fig. 1a). Neurons were then classified into the two groups based on the cluster analysis after PCA (Fig. 1b), and the average values for the properties measured were: adaptation index_C1_ = 17.7 ± 1.9; sAHP_C1_ = 2.7 ± 0.1 mV; % sag_C1_ = 10.2 ± 0.8%; and adaptation index_C2_ = 2.0 ± 0.3, *p* < 0.001; sAHP_C2_ = 1.2 ± 0.1 mV, *p* < 0.001; % sag_C2_ = 19.6 ± 0.9%, *p* < 0.001 (Fig. 1c–h). The co-occurrence of small sAHP, large sag, and small adaptation index in C2 and of large sAHP, small sag, and large adaptation index in C1 confirms previous reports^2–6,8–10^. As already suggested by the PC loadings, spike frequency adaptation index was the parameter with the largest fold change between clusters (~8.7 times larger in C1; Fig. 1f) and, therefore, we refer to cells in the cluster C1 as adapting while cells in the cluster C2 are referred to as non-adapting.

### Morphology of adapting and non-adapting L5 pyramidal neurons

To evaluate if the cells included in these two clusters exhibited morphological differences between them, a subset of neurons were filled with biocytin during whole-cell recordings. All the neurons filled showed the typical morphological features of L5 pyramidal neurons, i.e., pyramidal/triangular shape of the cell body and a prominent apical dendrite oriented towards and perpendicular to layer 1 (Fig. 1i). The morphological analysis of these neurons indicated that non-adapting neurons (C2) possessed significantly larger apical tufts and thicker apical dendrite shafts (Fig. 1i–k) and a larger number of primary apical dendrites terminating in a dendritic tuft near the pia (Fig. 1i,m) than adapting neurons. There was no difference in apical dendrite length between adapting and non-adapting neurons (Fig. 1l). Therefore, in our hands too, adapting cells belonged to the thin-tufted type and non-adapting neurons corresponded to the thick-tufted type (Fig. 1i–m). Our results confirm previous reports regarding the relationship between intrinsic electrophysiology and dendritic morphology, which indicated that large sAHP, small sag, and large adaptation index are preferentially found in thin-tufted cells, and small sAHP, large sag, and small adaptation index are preferentially found in thick-tufted cells^2,4,5,8^.

### Postsynaptic currents in adapting and non-adapting L5 pyramidal cells

**Figure 2:**
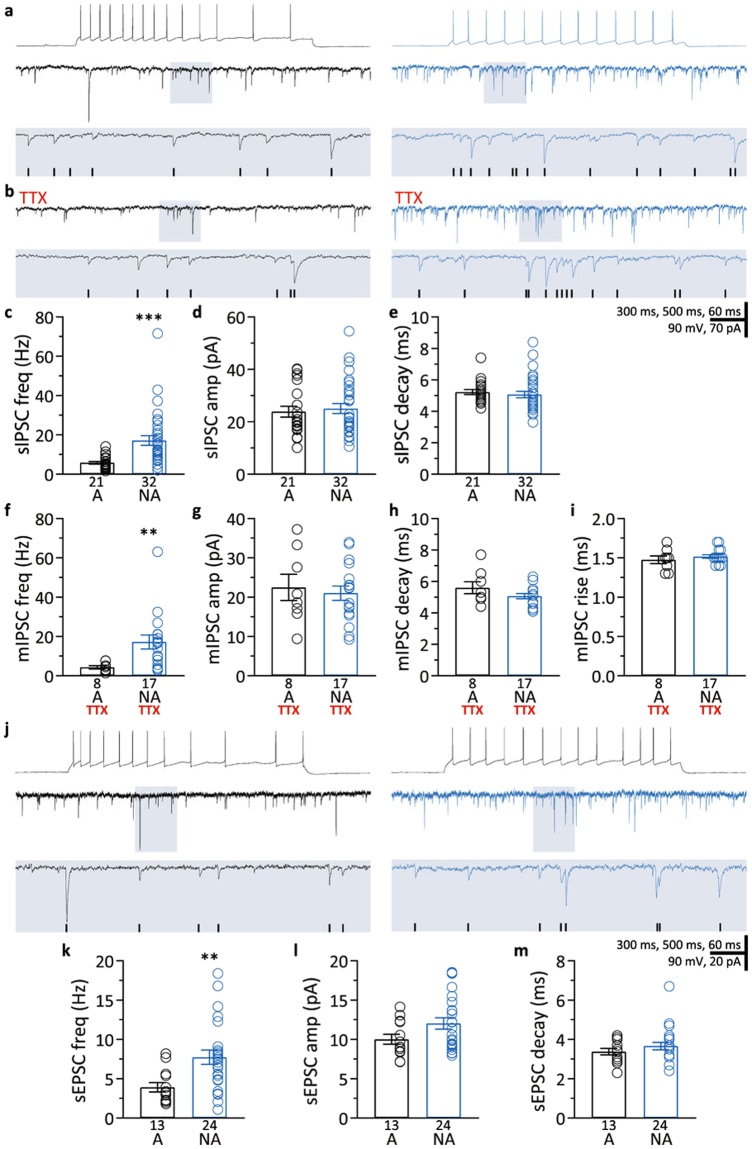
Characterization of synaptic inputs in adapting and non-adapting L5 pyramidal neurons. (**a**) Representative recordings illustrating lower sIPSC frequencies in an adapting neuron (left) than in a non-adapting neuron (right). Top traces, action potentials fired in response to intracellular current injection. Each middle trace shows a representative voltage-clamp recording of sIPSCs made from the neuron whose spiking is shown above. Lower traces are expansions of the shaded areas in the middle traces. Detected events are marked with black ticks in the shaded area. (**b**) Representative recordings showing lower mIPSC frequencies in the adapting neuron (left) than in the non-adapting neuron (right). Recordings are from the same neurons shown in (a). Lower traces are expansions of the shaded areas in the traces above. Black ticks represent detected events. mIPSCs were recorded while action potentials were blocked with 1 µM TTX. (**c–e**) Summary of sIPSC frequency (**c**), amplitude (**d**), and decay time (**e**) of sIPSCs recorded in adapting (A) and non-adapting (NA) neurons. (**f–i**) Summary of mIPSC frequency (**f**), amplitude (**g**), decay time (**h**), and rise time (**i**) of mIPSC recorded in adapting (A) and non-adapting (NA) neurons while action potentials were blocked with TTX. (**j**) Representative recordings illustrating lower sEPSC frequencies in an adapting neuron (left) than in a non-adapting neuron (right). Top traces, action potentials fired in response to intracellular current injection. Each middle trace shows a representative voltage-clamp recording of sEPSCs made from the neuron whose spiking is shown above. Lower traces are expansions of the shaded areas in the middle traces. Black ticks represent detected events. (**k–m**) Summary of sEPSC frequency (**k**), amplitude (**l**), and decay time (**m**) of sEPSCs recorded in adapting (A) and non-adapting (NA) neurons. Data presented as mean ± standard error. **p < 0.01; ***p < 0.001.

There are no detailed characterizations of spontaneous and miniature postsynaptic inputs in these two types of barrel cortex neurons available in the literature. However, this knowledge is critical for understanding information processing by the interaction of synaptic inputs and intrinsic properties. Moreover, such characterization can demonstrate that between-cell comparisons of synaptic inputs should be made according to cell subtype. For those reasons, once we identified adapting and non-adapting pyramidal neurons, we quantified their synaptic inputs. Given that changes in synaptic inhibition are hypothesized to occur in several neurological disorders^38–41^ and during aging^42–51^, we focused primarily on characterization of inhibitory postsynaptic currents. Comparing adapting neurons (C1) and non-adapting neurons (C2) revealed that the frequency of sIPSCs was approximately three-fold higher in non-adapting neurons (Fig. 2a,c and Table 1). We did not find a difference in sIPSC amplitude or decay time between the two groups (Fig. 2a,d,e and Table 1).

**Table 1:** sIPSC, mIPSC, and sEPSC frequency, amplitude, and decay time for adapting (A) and non-adapting (NA) neurons reported as mean ± standard error. Number in parentheses indicates sample size; *p < 0.01; ***p < 0.001.

	sIPSC		mIPSC		sEPSC	
	A (21)	NA (32)	A (8)	NA (17)	A (13)	NA (24)
Frequency (Hz)	5.9 ± 0.7	17.2 ± 2.5***	4.3 ± 0.8	17.2 ± 3.5*	3.9 ± 0.6	7.7 ± 0.9*
Amplitude (pA)	23.8 ± 2.0	25.0 ± 1.8	22.5 ± 3.3	21.0 ± 1.8	10.0 ± 0.6	12.1 ± 0.7
Decay (ms)	5.2 ± 0.2	5.1 ± 0.2	5.6 ± 0.4	5.1 ± 0.2	3.4 ± 0.2	3.7 ± 0.2

Are action potentials in the GABAergic neurons presynaptic to non-adapting neurons responsible for the higher IPSC frequencies in non-adapting neurons? To test this, after measuring sAHP, sag, and spike frequency adaptation index, we blocked action potentials with bath-applied TTX (1 μM), and subsequently recorded mIPSCs. Similar to sIPSCs, the frequency of mIPSCs was higher in non-adapting cells compared to adapting cells (Fig. 2b,f and Table 1). The mIPSC amplitude and decay time were not different between the two groups (Fig. 2b,g,h and Table 1). Likewise, the mIPSC rise times were not different (adapting neurons: 1.47 ± 0.05 ms; non-adapting neurons: 1.52 ± 0.02 ms, Fig. 2b,i). The within-cell comparison of IPSC frequency also showed no effect of action potential blockade, whether adapting and non-adapting cells were considered separately or pooled (*p* > 0.05, all paired *t*-tests, adapting *n* = 8; non-adapting *n* = 15). Both the lack of a TTX effect on IPSCs and the fact that mIPSCs, like sIPSCs, occurred at higher frequencies in non-adapting cells indicate that the higher frequency of IPSCs in non-adapting cells is not caused by higher levels of activity in the presynaptic GABAergic neurons. Taken together, our data on mIPSCs suggest that the higher frequency of IPSCs in non-adapting neurons is most likely caused by a larger number of GABAergic synapses on these neurons. On the other hand, the lack of difference in amplitude, rise time, and decay time in mIPSCs suggest that GABAergic innervation is similarly distributed along the somatodendritic axis in adapting and non-adapting neurons, at least when assessed from synaptic currents recorded at the soma.

Although our study focused on IPSCs, we also surveyed sEPSCs to gain a first understanding of the EPSC/IPSC balance in adapting and non-adapting barrel cortex L5 pyramidal neurons. We found that non-adapting neurons had a higher frequency of sEPSCs than adapting neurons (Fig. 2j–m and Table 1). On the other hand, sEPSC amplitude and decay time were not different between the two types of neurons. Furthermore, while the mean sEPSC/sIPSC frequency ratio for adapting cells was 0.67, the non-adapting cells’ sEPSC/sIPSC ratio was 0.45, indicating that non-adapting L5 pyramidal neurons receive 33% fewer excitatory currents per inhibitory current. Our results show that while non-adapting cells receive higher frequencies of both IPSCs and EPSCs than adapting cells, the excitation/inhibition balance in adapting cells is shifted towards excitation due to a proportional higher frequency of EPSCs per IPSC.

### Intrinsic properties and synaptic inputs of thy1-GFP L5 pyramidal neurons

**Figure 3:**
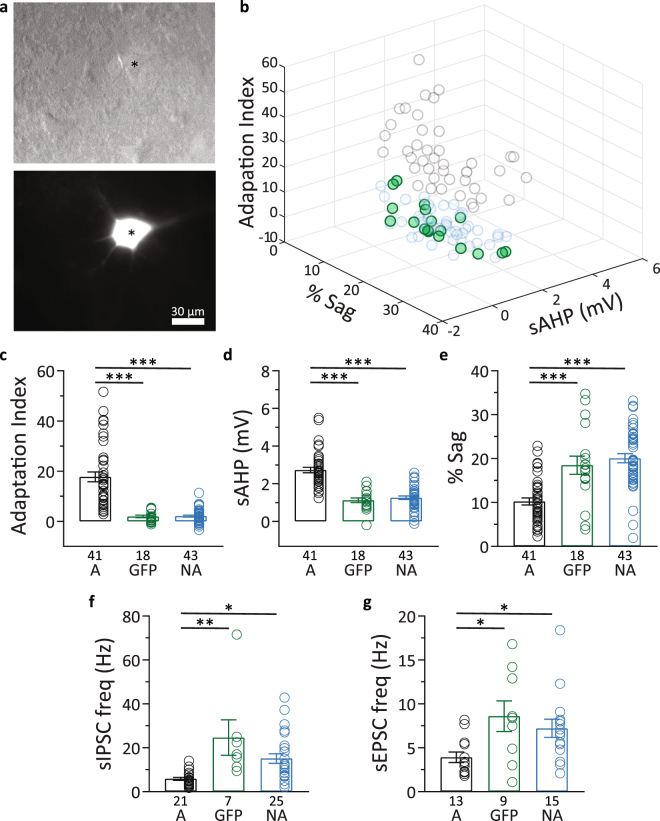
Intrinsic properties, sIPSCs, and sEPSCs in thy1-GFP+ neurons. (**a**) Top, representative example of a L5 pyramidal neuron in S1BF under Dodt gradient contrast (*). Bottom, epifluorescence image of the same field of view showing the “*”denoted neuron is GFP+. (**b**) 3D scatter plot of adapting and non-adapting pyramidal neurons as in Fig. 1b, highlighting the thy-1 GFP+ neurons (green circles) included in the cluster analysis. (**c–e**) Summary of data showing that GFP+ cells (GFP) and GFP− cells from the non-adapting group (NA) did not differ from each other in adaptation index (**c**), sAHP (**d**), and sag (**e**) but both of these cell groups had different sAHP, sag, and adaptation index compared to adapting L5 pyramidal neurons (A). (**f**) Summary of data showing that GFP+ cells (GFP) and GFP− cells from the non-adapting group (NA) did not differ in sIPSC frequency, but both had higher sIPSC frequencies compared to adapting L5 pyramidal neurons (A). (**g**) Summary of data showing that GFP+ cells (GFP) and GFP− cells from the non-adapting group (NA) did not differ in sEPSC frequency, but both had higher sEPSC frequencies compared to adapting L5 pyramidal neurons (A). None of the adapting cells in this study were GFP+. Data presented as mean ± standard error. *p < 0.05; **p < 0.01; ***p < 0.001.

Transgenic mice carrying thy-1 gene regulatory elements are used extensively in imaging studies due to the sparse expression pattern of fluorophores achieved in the neocortex^36,52–54^. Several neurological disorder^55–57^ and aging^58^ studies use these transgenic mice to monitor aberrant structural and functional changes involving dendritic spine morphology and dynamics. Inhibitory neurotransmission has been hypothesized to modulate these dendritic spine properties^59–61^. Therefore, we wanted to characterize the intrinsic electrophysiology and inhibitory synaptic inputs of the barrel cortex L5 thy1-GFP neurons. We recorded from both GFP+ and GFP− cells, and found that all 18 GFP+ cells belonged to the non-adapting cluster C2 (Fig. 3a,b). GFP+ cells and GFP− cells from the non-adapting group did not differ from each other in sAHP, sag, and adaptation index, but both of these cell groups had different sAHP, sag, and adaptation index compared to adapting L5 pyramidal neurons (Fig. 3b–e and Table 2).

**Table 2:** The values of the intrinsic electrophysiology parameters adaptation index, sAHP, and sag for adapting neurons (A), GFP+ neurons (GFP), and GFP− non-adapting neurons (NA) reported as mean ± standard error.

	A (41)	NA GFP+ (18)	NA GFP− (43)
Adaptation index	17.7 ± 1.9	2.0 ± 0.5***	2.1 ± 0.4***
sAHP (mV)	2.7 ± 0.1	1.1 ± 0.1***	1.3 ± 0.1***
% sag	10.2 ± 0.8	18.5 ± 2.1***	20.1 ± 1.0***

None of the adapting cells in this study were GFP+. Number in parentheses indicates sample size; ***p < 0.001 vs. adapting neurons (A).

GFP+ and GFP− cells from the non-adapting group did not differ in sIPSC frequency, but both had higher sIPSC frequencies compared to adapting L5 pyramidal neurons in C1 (Fig. 3f and Table 3). sIPSC amplitude and decay time were not different in the three groups of cells (Table 3). We also extended our experiment to recordings of excitatory inputs, and found that GFP+ neurons and GFP− non-adapting pyramidal neurons did not differ in sEPSC frequency, but both of these cell groups had a higher sEPSC frequency than adapting neurons (Fig. 3g and Table 3). As was the case for sIPSCs, we found no difference in sEPSC amplitude or decay time between the three groups of cells (Table 3). Our data show that thy1-GFP neurons in L5 S1BF are non-adapting, in accord with previous reports on thy1-YFP neurons^9,62^. Like GFP− non-adapting neurons, L5 thy1-GFP neurons have higher sIPSC and sEPSC frequencies than adapting L5 pyramidal neurons.

**Table 3:** sIPSC and sEPSC frequency, amplitude, and decay time for adapting neurons (A), GFP+ neurons (GFP), and GFP− non-adapting neurons (NA) reported as mean ± standard error. None of the adapting cells in this study were GFP+. Number in parentheses indicates sample size; *p < 0.05and **p < 0.01 vs. adapting neurons (A).

	sIPSC			sEPSC		
	A (21)	NA GFP+ (7)	NA GFP− (25)	A (13)	NA GFP+ (9)	NA GFP− (15)
Frequency (Hz)	5.9 ± 0.7	24.7 ± 8.1**	15.1 ± 2.2*	3.9 ± 0.60	8.6 ± 1.7*	7.2 ± 1.0*
Amplitude (pA)	23.8 ± 2.0	18.6 ± 2.5	26.9 ± 2.1	10.0 ± 0.6	11.8 ± 1.0	12.1 ± 1.0
Decay (ms)	5.2 ± 0.2	4.8 ± 0.2	5.2 ± 0.2	3.4 ± 0.2	3.5 ± 0.2	3.8 ± 0.3

### Synaptic inputs to S1BF L5 pyramidal neurons of female and male mice

Because we used both female and male mice we asked if there were gender-specific differences in the frequency of sIPSCs or sEPSCs in either adapting or non-adapting L5 pyramidal neurons. However, this was not the case. There was no difference in adapting cells’ sIPSCs (6.8 ± 1.1 vs. 4.8 ± 0.6 Hz; *n* = 11, 10 cells; *p* > 0.05), non-adapting cells’ sIPSCs (17.2 ± 2.7 vs. 17.2 ± 4.6 Hz; *n* = 18, 14 cells; *p* > 0.05), adapting cells’ sEPSCs (4.1 ± 1.2 vs. 3.8 ± 0.7 Hz; *n* = 5, 8 cells; *p* > 0.05), or non-adapting cells’ sEPSCs (8.8 ± 1.3 vs. 6.2 ± 1.1 Hz; *n* = 14, 10 cells; *p* > 0.05) between female and male mice, respectively.

## Discussion

Two main subtypes of L5 pyramidal neurons have been established based on their projections: intratelencephalic (IT) neurons, whose axons stay in the ipsi- or contralateral cortex and/or the striatum, and pyramidal tract-type (PT) neurons, whose axonal branches project to the ipsilateral cortex and striatum and beyond the telencephalon^2,4–6,9,10,62^. While the intrinsic properties and morphological features of these two types of neurons have been extensively described and used for further subdivision into additional subtypes of L5 neurons^4,7,63^, little is known about the frequency, amplitude, and balance of spontaneous excitatory and inhibitory synaptic inputs these neurons receive. In this study we used unsupervised clustering analysis after PCA of three intrinsic properties (sAHP, sag, and spike frequency adaptation index) of S1BF L5 pyramidal neurons and identified two clusters of neurons that morphologically and electrophysiologically conform to the aforementioned IT and PT neuron types. We characterized the synaptic inputs to neurons from these two clusters, which we refer to as adapting and non-adapting neurons, and that correspond to the IT and PT subtypes, respectively. We found that non-adapting L5 pyramidal neurons receive higher frequency of both, sIPSCs and sEPSCs, than adapting neurons. Furthermore, the sEPSC/sIPSC frequency ratio was ~33% lower in non-adapting neurons, indicating that the E/I balance is shifted to favor inhibition over excitation in non-adapting L5 pyramidal neurons compared with adapting neurons.

Similarly to previously published studies, spike frequency adaptation proved to be an appropriate parameter to catalogue cells residing in L5. In fact, adaptation index and sag, while negatively correlated, were the parameters that contributed the most to the principal component and therefore had the largest influence on the final clustering of the cells (Fig. 1a,b). The co-occurrence of small adaptation index, large sag, and small sAHP in non-adapting and of large adaptation index, small sag, and large sAHP in adapting L5 pyramidal neurons is in agreement with previous reports^2,4,6,8^. In addition, the analysis of morphological features showed that adapting neurons present thinner primary apical dendrites and less elaborated dendritic arbors than non-adapting neurons. Our results corroborate previous studies indicating that large adaptation index, small sag, and large sAHP are preferentially found in adapting thin-tufted cells, and small adaptation index, large sag, and small sAHP, are preferentially found in non-adapting thick-tufted cells^2,4,5,8^.

The characterization of synaptic inputs showed that the amplitude and decay of sIPSCs and sEPSCs were comparable between these two types of L5 pyramidal neurons. However non-adapting neurons presented higher frequencies of both sIPSCs and sEPSCs. The difference in IPSC frequency that we observed is maintained during action potential blockade, matching results from genetically labeled neurons in L5 of the primary motor cortex of mice and from neurons classified by the presence or absence of burst firing in L5 of the primary auditory cortex of rats^64,65^. A possible cause of the elevated mIPSC frequency in non-adapting neurons is a higher number of GABAergic synapses on these cells, which have been shown to have wider dendritic tufts with a dendritic area significantly larger than that of thin-tufted neurons^1,10,66,67^, consistent with our results (Fig. 1i,j). The inhibitory microcircuit organization has been proposed as a key factor in the differential activity regulation of the main two subtypes of L5 pyramidal neurons by particular interneuron types^68^ and it may explain the different mIPSC frequencies in adapting and non-adapting pyramidal neurons found in our study. While intrinsic-bursting corticofugal L5 pyramidal neurons receive mostly thalamic-driven inhibitory inputs from fast-spiking parvalbumin interneurons, regular-spiking corticocortico L5 neurons receive mostly intracortical-driven inhibition likely from interneurons other than fast-spiking neurons^68^. Another potential source of difference in the IPSC frequency reported here could be different release probabilities from GABAergic neurons presynaptic to adapting and non-adapting neurons. This hypothesis is intrinsically linked to and difficult to separate from the notion that different types of interneurons, such as fast-spiking and somatostatin-expressing neurons with high and relatively low probability of release, respectively, preferentially innervate different types of pyramidal cells^66,69–72^. The higher frequency of IPSCs in non-adapting neurons may provide a more extensive substrate for information processing in these cells, and a higher number of GABAergic synapses would provide more opportunities for plasticity. Moreover, the higher level of inhibition also enables a larger range of inhibition for situations requiring robust shifts in activity.

Similarly, the higher frequency of sEPSCs detected in non-adapting neurons could be explained by the fact that these neurons have more complex dendritic arbors (Fig. 1i,j) and by synaptic inputs coming from neurons with different firing rates, i.e., thalamic inputs predominantly projecting onto non-adapting neurons *versus* intracortical inputs onto adapting neurons, as previously suggested^68,73^. Interestingly, the differences in sIPSC and sEPSC frequencies between adapting and non-adapting neurons were not equally proportional, resulting in different E/I ratios. While adapting L5 pyramidal neurons presented a mean sEPSC/sIPSC frequency ratio of 0.68, non-adapting neurons presented a mean E/I ratio of 0.45, an approximately 33% lower E/I ratio than adapting neurons.

There is no theoretical reason that we are aware of that would suggest that the elevated IPSC frequency will lead to a higher adaptation index. However, as it has been suggested, the higher frequency of EPSCs in non-adapting neurons may result in a homeostatic upregulation of the hyperpolarization–activated current (I_h_) that diminishes excitability in response to higher frequencies of EPSCs^16^, and therefore keeps spike frequency more constant. This possible role of I_h_ in non-adapting neurons is supported by reports describing: 1) the expression of larger numbers of HCN channels in the more extensive dendritic arbors of corticospinal thick-tufted neurons^17^; 2) no spike frequency adaptation and a large I_h_ mediating the large sag potentials observed in corticocollicular L5 pyramidal neurons in auditory cortex^74^; and 3) increased L2/3-driven spiking after inhibition of I_h_ in L5 corticospinal neurons but not in corticostriatal neurons in motor cortex^17^.

The thy1-GFP-M mouse line as well as many other lines of transgenic mice derived from the same thy-1 construct^36^ are widely used for *in vivo* imaging of neuronal structure and function and optogenetic studies due to the intense, yet sparse labeling of specific subsets of neurons^52,58,75,76^. These mouse lines present a robust expression of fluorophores like GFP and YFP, calcium sensors like GCaMP, or light-gated ion channels like channelrhodopsin throughout L5 pyramidal neurons of the cortex. Our data indicate, however, that the expression of GFP seems to be restricted to non-adapting L5 neurons. It is important then to characterize the intrinsic properties of this subtype of thy1-GFP+ pyramidal neurons and the inputs to them to better understand and interpret the results from imaging and electrophysiology studies targeting these neurons. Our results from thy1-GFP+ neurons: 1) confirm previously reported data from thy1 neurons^9,62^, indicating their PT-like, non-adapting, nature; 2) provide detailed characterization of the intrinsic properties of this subtype of pyramidal neuron; and 3) describe the significantly higher frequency of IPSCs and EPSCs in these neurons compared with adapting pyramidal neurons from the same cortical layer.

In summary, our data reveal striking differences in the frequency of synaptic inputs, both excitatory and inhibitory, between adapting and non-adapting L5 pyramidal neurons in S1BF, suggesting the need to identify pyramidal neurons’ subtype in any between-cell comparisons of IPSC or EPSC frequency. These differences in synaptic input frequency may also have possible implications for the excitation/inhibition balance, an important determinant of neuronal activity. A loss of comparable absolute levels of inhibition in adapting and non-adapting cells would leave a larger unbalanced excitatory current in adapting cells. Likewise, a comparable increase in inhibition in the two cell types will leave a larger unbalanced inhibitory current in adapting cells. Thus, L5 pyramidal neurons of different subtype and function may have their excitation/inhibition balance of synaptic inputs differentially disturbed by drugs which boost GABAergic inputs, such as benzodiazepines, in conditions where brain oscillations are altered, or in conditions impairing inhibitory synaptic transmission.
