# Publisher Correction: Marked bias towards spontaneous synaptic inhibition distinguishes non-adapting from adapting layer 5 pyramidal neurons in the barrel cortex

**DOI:** 10.1038/s41598-017-18708-w

**Published:** 2018-01-15

**Authors:** Ion R. Popescu, Kathy Q. Le, Rocío Palenzuela, Rebecca Voglewede, Ricardo Mostany

**Affiliations:** 1 Department of Pharmacology, Tulane University School of Medicine, New Orleans, 70112 USA; 2 Neuroscience Program, Brain Institute, Tulane University, New Orleans, 70118 USA; 3 School of Experimental Sciences, Universidad Francisco de Vitoria, Pozuelo de Alarcón, 28223 Madrid, Spain; 4 Brain Institute, Tulane University, New Orleans, 70118 USA

Correction to: *Scientific Reports* 10.1038/s41598-017-14971-z, published online 02 November 2017

The original PDF version of this Article contained an error where Figure 2 was truncated. The correct figure appears below as Figure 1. This has now been corrected in the PDF; the HTML version of the paper was correct from the time of publication.

**Figure 1:**
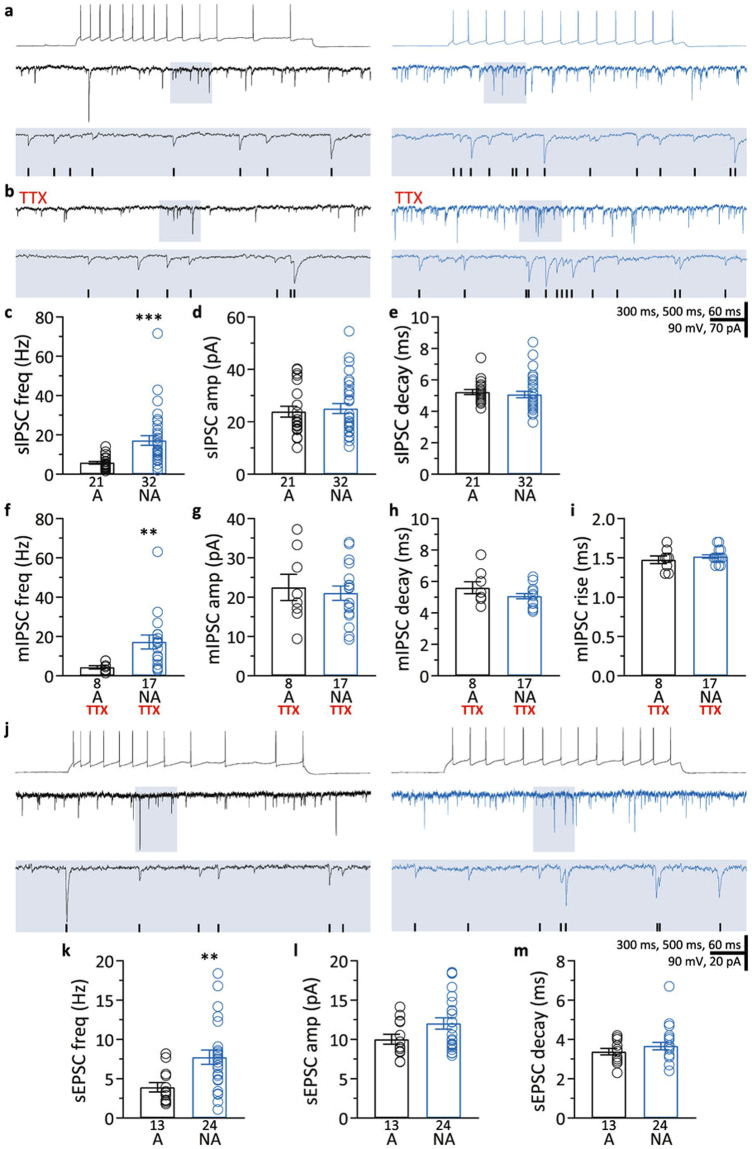
.

